# 4-Amino-3-ammonio­pyridinium dinitrate

**DOI:** 10.1107/S1600536810005556

**Published:** 2010-02-17

**Authors:** Madhukar Hemamalini, Hoong-Kun Fun

**Affiliations:** a X-ray Crystallography Unit, School of Physics, Universiti Sains Malaysia, 11800 USM, Penang, Malaysia

## Abstract

In the crystal structure of the title compound, C_5_H_9_N_3_^2+^·2NO_3_^−^, the cations and anions are connected by inter­molecular N—H⋯O and C—H⋯O hydrogen bonds, forming a three-dimensional network. The crystal structure is further stabilized by π⋯π inter­actions between pyridinium rings [centroid–centroid distance = 3.775 (4) Å].

## Related literature

For background to the chemistry of substituted pyridines, see: Pozharski *et al.* (1997 ▶); Katritzky *et al.* (1996 ▶); Abu Zuhri & Cox (1989 ▶). For related structures, see: Fun & Balasubramani (2009 ▶); Rubin-Preminger & Englert (2007 ▶); Qin & Wang (2009 ▶). For details of hydrogen bonding, see: Jeffrey & Saenger (1991 ▶); Jeffrey (1997 ▶); Scheiner (1997 ▶). For reference bond-length data, see: Allen *et al.* (1987 ▶). For the stability of the temperature controller used in the data collection, see: Cosier & Glazer (1986 ▶).
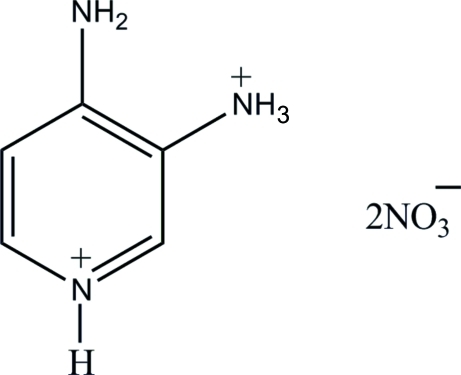


## Experimental

### 

#### Crystal data

C_5_H_9_N_3_^2+^·2NO_3_^−^
*M* *_r_* = 235.17
Monoclinic, 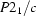

*a* = 12.3008 (5) Å
*b* = 10.5086 (5) Å
*c* = 7.1411 (3) Å
β = 97.546 (1)°
*V* = 915.09 (7) Å^3^
*Z* = 4
Mo *K*α radiation
μ = 0.16 mm^−1^
*T* = 100 K
0.65 × 0.37 × 0.28 mm

#### Data collection

Bruker APEX DUO CCD area-detector diffractometer
Absorption correction: multi-scan (*SADABS*; Bruker, 2009 ▶) *T*_min_ = 0.906, *T*_max_ = 0.958
18234 measured reflections
4796 independent reflections
4129 reflections with *I* > 2σ(*I*)
*R*_int_ = 0.024

#### Refinement

*R*[*F* ^2^ > 2σ(*F* ^2^)] = 0.033
*wR*(*F* ^2^) = 0.096
*S* = 1.06
4796 reflections
181 parameters
All H-atom parameters refined
Δρ_max_ = 0.66 e Å^−3^
Δρ_min_ = −0.26 e Å^−3^

### 

Data collection: *APEX2* (Bruker, 2009 ▶); cell refinement: *SAINT* (Bruker, 2009 ▶); data reduction: *SAINT*; program(s) used to solve structure: *SHELXTL* (Sheldrick, 2008 ▶); program(s) used to refine structure: *SHELXTL*; molecular graphics: *SHELXTL*; software used to prepare material for publication: *SHELXTL* and *PLATON* (Spek, 2009 ▶).

## Supplementary Material

Crystal structure: contains datablocks global, I. DOI: 10.1107/S1600536810005556/rz2419sup1.cif

Structure factors: contains datablocks I. DOI: 10.1107/S1600536810005556/rz2419Isup2.hkl

Additional supplementary materials:  crystallographic information; 3D view; checkCIF report

## Figures and Tables

**Table 1:** Hydrogen-bond geometry (Å, °)

*D*—H⋯*A*	*D*—H	H⋯*A*	*D*⋯*A*	*D*—H⋯*A*
N1—H1*N*1⋯O2^i^	0.874 (13)	2.001 (13)	2.7750 (7)	147.0 (12)
N2—H1*N*2⋯O5	0.935 (14)	2.105 (15)	2.9070 (8)	143.1 (12)
N2—H1*N*2⋯O2^ii^	0.935 (14)	2.211 (14)	2.7767 (7)	118.1 (11)
N2—H2*N*2⋯O3^iii^	0.881 (14)	2.193 (14)	3.0006 (7)	152.3 (12)
N2—H2*N*2⋯O3^iv^	0.881 (14)	2.482 (14)	2.9231 (7)	111.6 (11)
N3—H1*N*3⋯O4	0.844 (16)	2.054 (16)	2.8653 (8)	161.0 (14)
N3—H2*N*3⋯O6^v^	0.827 (12)	2.130 (12)	2.9442 (7)	168.0 (12)
N2—H3*N*2⋯O5^iv^	0.871 (12)	1.963 (12)	2.8227 (8)	169.0 (12)
N2—H3*N*2⋯O6^iv^	0.871 (12)	2.494 (12)	3.1217 (7)	129.5 (10)
C2—H2⋯O3^iii^	0.910 (11)	2.439 (11)	3.0489 (8)	124.6 (9)
C2—H2⋯O1^vi^	0.910 (11)	2.552 (11)	3.1834 (8)	127.0 (9)
C2—H2⋯O3^iv^	0.910 (11)	2.570 (11)	3.1277 (8)	120.2 (9)
C3—H3⋯O6^i^	0.979 (12)	2.253 (12)	3.1170 (8)	146.6 (10)
C4—H4⋯O4^v^	0.926 (12)	2.559 (12)	3.4274 (8)	156.3 (10)

Symmetry codes: (i) 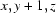; (ii) 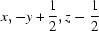; (iii) 
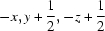
; (iv) 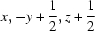; (v) 
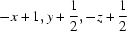
; (vi) 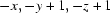.

**Fig. 1.**
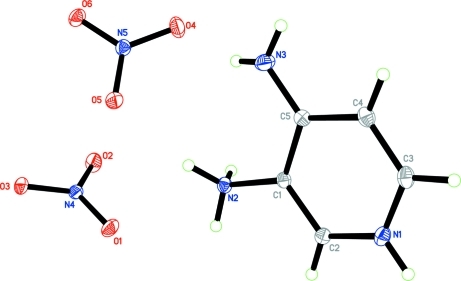
The asymmetric unit of the title compound. Displacement ellipsoids are drawn at the 50% probability level.

**Fig. 2.**
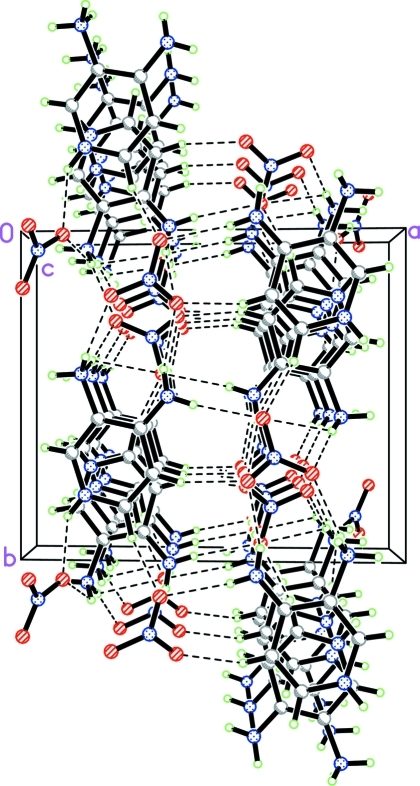
The crystal packing of the title compound, showing hydrogen-bonded (dashed lines) networks.

## supplementary crystallographic information

### Comment

Pyridine and its derivatives play an important role in heterocyclic chemistry (Pozharski *et al.*, 1997; Katritzky *et al.*, 1996). In particular, diaminopyridines play an important role in the preparation of aromatic azo dyes, the subject of many polarographic investigations (Abu Zuhri & Cox, 1989). Pyridine and its substituted derivatives are often involved in hydrogen-bond interactions (Jeffrey & Saenger, 1991; Jeffrey, 1997; Scheiner, 1997).  The crystal structures of 3,4-diaminopyridine (Rubin-Preminger & Englert, 2007), 3,4-diaminopyridinium hydrogen succinate (Fun & Balasubramani, 2009) and 4-amino-3-ammoniopyridinium dichloride (Qin & Wang, 2009) have been reported in the literature. In order to study some interesting hydrogen bonding interactions, the synthesis and structure of the title salt is presented here.

The asymmetric unit of the title compound (Fig. 1) consists of a diprotonated 3,4-diaminopyridine cation and two nitrate anions. In the 3,4-diaminopyridinium cation, protonation at atom N1 has lead to a slight increase in the C2—N1—C3 angle to 121.45 (5)° compared to those of an unprotonated structure (Rubin-Preminger & Englert, 2007).  The 3-amino N atom (N2) is also protonated.  This type of protonation has also been observed in the crystal structure of 4-amino-3-ammoniopyridinium dichloride (Qin & Wang, 2009). The bond lengths (Allen *et al.*, 1987) and angles are normal.

In the crystal structure (Fig. 2), the anions and cations are connected by intermolecular strong N—H···O and weak C—H···O hydrogen bonds, forming a three-dimensional network.  The crystal structure is further stabilized by π···π interactions between the pyridinium rings (N1/C1–C5) [centroid-to-centroid (x, 3/2-y, -1/2+z and x, 3/2-y, 1/2+z) distance = 3.775 (4)Å].

### Experimental

To a hot methanol solution (20 ml) of 3,4-diaminopyridine (27 mg, Aldrich) was added a few drops of nitric acid. The solution was warmed over a water bath for a few minutes. The resulting solution was allowed to cool slowly to room temperature. Crystals of the title compound appeared from the mother liquor after a few days.

### Refinement

All the H atoms were located in a difference Fourier map and allowed to refine freely [N—H = 0.827 (13) - 0.934 (15)Å, C—H = 0.91 (11) - 0.978 (12) Å].

### Crystal data

**Table:**

C_5_H_9_N_3_^2+^·2NO^3^^−^	*F*(000) = 488
*M**_r_* = 235.17	*D*_x_ = 1.707 Mg m^−^^3^
Monoclinic, *P*2_1_/*c*	Mo *K*α radiation, λ = 0.71073 Å
Hall symbol: -P 2ybc	Cell parameters from 9066 reflections
*a* = 12.3008 (5) Å	θ = 3.3–37.5°
*b* = 10.5086 (5) Å	µ = 0.16 mm^−^^1^
*c* = 7.1411 (3) Å	*T* = 100 K
β = 97.546 (1)°	Block, colourless
*V* = 915.09 (7) Å^3^	0.65 × 0.37 × 0.28 mm
*Z* = 4	

### Data collection

**Table:**

Bruker APEX DUO CCD area-detector diffractometer	4796 independent reflections
Radiation source: fine-focus sealed tube	4129 reflections with *I* > 2σ(*I*)
graphite	*R*_int_ = 0.024
φ and ω scans	θ_max_ = 37.6°, θ_min_ = 1.7°
Absorption correction: multi-scan (*SADABS*; Bruker, 2009)	*h* = −21→20
*T*_min_ = 0.906, *T*_max_ = 0.958	*k* = −17→17
18234 measured reflections	*l* = −10→12

### Refinement

**Table:**

Refinement on *F*^2^	Primary atom site location: structure-invariant direct methods
Least-squares matrix: full	Secondary atom site location: difference Fourier map
*R*[*F*^2^ > 2σ(*F*^2^)] = 0.033	Hydrogen site location: inferred from neighbouring sites
*wR*(*F*^2^) = 0.096	All H-atom parameters refined
*S* = 1.06	*w* = 1/[σ^2^(*F*_o_^2^) + (0.0545*P*)^2^ + 0.1242*P*] where *P* = (*F*_o_^2^ + 2*F*_c_^2^)/3
4796 reflections	(Δ/σ)_max_ = 0.001
181 parameters	Δρ_max_ = 0.66 e Å^−^^3^
0 restraints	Δρ_min_ = −0.26 e Å^−^^3^

### Special details

**Table:**

Experimental. The crystal was placed in the cold stream of an Oxford Cryosystems Cobra open-flow nitrogen cryostat (Cosier & Glazer, 1986) operating at 100.0 (1) k.
Geometry. All s.u.'s (except the s.u. in the dihedral angle between two l.s. planes) are estimated using the full covariance matrix. The cell s.u.'s are taken into account individually in the estimation of s.u.'s in distances, angles and torsion angles; correlations between s.u.'s in cell parameters are only used when they are defined by crystal symmetry. An approximate (isotropic) treatment of cell s.u.'s is used for estimating s.u.'s involving l.s. planes.
Refinement. Refinement of F^2^ against ALL reflections. The weighted R-factor wR and goodness of fit S are based on F^2^, conventional R-factors R are based on F, with F set to zero for negative F^2^. The threshold expression of F^2^ > 2σ(F^2^) is used only for calculating R-factors(gt) etc. and is not relevant to the choice of reflections for refinement. R-factors based on F^2^ are statistically about twice as large as those based on F, and R- factors based on ALL data will be even larger.

### Fractional atomic coordinates and isotropic or equivalent isotropic displacement parameters (Å^2^)

**Table:**

	*x*	*y*	*z*	*U*_iso_*/*U*_eq_	
N1	0.18671 (4)	0.78615 (5)	0.42895 (7)	0.01262 (9)	
N2	0.17802 (4)	0.43778 (5)	0.39491 (7)	0.01067 (8)	
N3	0.39285 (4)	0.50229 (5)	0.29631 (9)	0.01537 (10)	
C1	0.21985 (4)	0.56747 (5)	0.39298 (8)	0.00969 (9)	
C2	0.15309 (5)	0.66472 (5)	0.43549 (8)	0.01097 (9)	
C3	0.28652 (5)	0.81587 (6)	0.38180 (9)	0.01366 (10)	
C4	0.35654 (5)	0.72296 (6)	0.33967 (9)	0.01302 (10)	
C5	0.32559 (5)	0.59285 (5)	0.34337 (8)	0.01084 (9)	
N4	0.07181 (4)	0.08353 (5)	0.36523 (7)	0.01101 (8)	
O1	0.03294 (4)	0.19166 (5)	0.37605 (8)	0.01831 (10)	
O2	0.12811 (4)	0.03373 (5)	0.50656 (7)	0.01704 (9)	
O3	0.05534 (4)	0.02013 (5)	0.21333 (7)	0.01419 (8)	
N5	0.34927 (4)	0.17325 (5)	0.27032 (7)	0.01208 (9)	
O4	0.41357 (4)	0.23657 (5)	0.38374 (8)	0.01818 (9)	
O5	0.25728 (4)	0.21885 (5)	0.20404 (8)	0.01714 (9)	
O6	0.37509 (4)	0.06465 (4)	0.21848 (7)	0.01541 (9)	
H2	0.0849 (9)	0.6508 (11)	0.4677 (16)	0.015 (2)*	
H3	0.3044 (10)	0.9065 (11)	0.3781 (17)	0.019 (3)*	
H4	0.4245 (10)	0.7452 (11)	0.3072 (17)	0.018 (2)*	
H1N1	0.1431 (11)	0.8483 (12)	0.4515 (19)	0.024 (3)*	
H1N2	0.1923 (12)	0.3916 (14)	0.289 (2)	0.036 (3)*	
H2N2	0.1064 (11)	0.4422 (13)	0.393 (2)	0.030 (3)*	
H1N3	0.3832 (12)	0.4256 (15)	0.325 (2)	0.036 (4)*	
H2N3	0.4547 (10)	0.5255 (11)	0.2771 (18)	0.022 (3)*	
H3N2	0.2027 (10)	0.3986 (12)	0.4994 (17)	0.022 (3)*	

### Atomic displacement parameters (Å^2^)

**Table:**

	*U*^11^	*U*^22^	*U*^33^	*U*^12^	*U*^13^	*U*^23^
N1	0.0158 (2)	0.00975 (18)	0.01202 (19)	0.00126 (15)	0.00078 (15)	−0.00025 (14)
N2	0.01164 (19)	0.00934 (17)	0.01122 (19)	−0.00130 (14)	0.00219 (15)	−0.00007 (14)
N3	0.0131 (2)	0.0132 (2)	0.0209 (2)	0.00103 (16)	0.00640 (18)	0.00051 (17)
C1	0.01061 (19)	0.00874 (19)	0.00967 (19)	−0.00068 (14)	0.00112 (15)	0.00042 (15)
C2	0.0119 (2)	0.0106 (2)	0.0102 (2)	0.00047 (15)	0.00092 (16)	0.00022 (15)
C3	0.0177 (2)	0.0110 (2)	0.0120 (2)	−0.00248 (17)	0.00083 (18)	0.00069 (17)
C4	0.0137 (2)	0.0124 (2)	0.0130 (2)	−0.00291 (17)	0.00193 (17)	0.00085 (16)
C5	0.0109 (2)	0.0114 (2)	0.0102 (2)	−0.00046 (15)	0.00145 (16)	0.00062 (16)
N4	0.01104 (18)	0.01129 (18)	0.01079 (18)	0.00072 (14)	0.00182 (14)	−0.00057 (14)
O1	0.0205 (2)	0.01273 (19)	0.0212 (2)	0.00626 (15)	0.00077 (17)	−0.00324 (15)
O2	0.0227 (2)	0.0157 (2)	0.01131 (18)	0.00386 (16)	−0.00316 (16)	0.00055 (14)
O3	0.01560 (19)	0.01632 (19)	0.01055 (17)	0.00110 (14)	0.00129 (14)	−0.00395 (14)
N5	0.01301 (19)	0.01063 (18)	0.0129 (2)	−0.00098 (14)	0.00270 (15)	−0.00080 (14)
O4	0.0181 (2)	0.0161 (2)	0.0189 (2)	−0.00316 (15)	−0.00271 (16)	−0.00424 (16)
O5	0.01401 (19)	0.0165 (2)	0.0200 (2)	0.00356 (15)	−0.00103 (15)	−0.00464 (16)
O6	0.0170 (2)	0.00960 (17)	0.0202 (2)	0.00105 (13)	0.00446 (16)	−0.00208 (14)

### Geometric parameters (Å, °)

**Table:**

N1—C2	1.3442 (7)	C2—H2	0.910 (11)
N1—C3	1.3516 (8)	C3—C4	1.3615 (9)
N1—H1N1	0.873 (13)	C3—H3	0.978 (12)
N2—C1	1.4575 (7)	C4—C5	1.4205 (8)
N2—H1N2	0.934 (15)	C4—H4	0.926 (12)
N2—H2N2	0.881 (14)	N4—O1	1.2392 (7)
N2—H3N2	0.871 (13)	N4—O2	1.2603 (7)
N3—C5	1.3327 (8)	N4—O3	1.2664 (7)
N3—H1N3	0.844 (16)	N5—O4	1.2474 (7)
N3—H2N3	0.827 (13)	N5—O6	1.2534 (7)
C1—C2	1.3698 (8)	N5—O5	1.2623 (7)
C1—C5	1.4176 (8)		
			
C2—N1—C3	121.45 (5)	C1—C2—H2	122.4 (7)
C2—N1—H1N1	120.3 (9)	N1—C3—C4	120.74 (5)
C3—N1—H1N1	118.3 (9)	N1—C3—H3	116.5 (7)
C1—N2—H1N2	111.9 (9)	C4—C3—H3	122.7 (7)
C1—N2—H2N2	107.7 (9)	C3—C4—C5	120.48 (5)
H1N2—N2—H2N2	108.1 (13)	C3—C4—H4	119.5 (7)
C1—N2—H3N2	111.5 (8)	C5—C4—H4	120.1 (7)
H1N2—N2—H3N2	111.5 (12)	N3—C5—C1	123.30 (5)
H2N2—N2—H3N2	105.8 (12)	N3—C5—C4	120.39 (5)
C5—N3—H1N3	120.6 (10)	C1—C5—C4	116.28 (5)
C5—N3—H2N3	116.5 (8)	O1—N4—O2	120.45 (5)
H1N3—N3—H2N3	118.9 (13)	O1—N4—O3	121.07 (5)
C2—C1—C5	120.77 (5)	O2—N4—O3	118.47 (5)
C2—C1—N2	118.22 (5)	O4—N5—O6	120.92 (5)
C5—C1—N2	120.99 (5)	O4—N5—O5	120.11 (5)
N1—C2—C1	120.29 (5)	O6—N5—O5	118.97 (5)
N1—C2—H2	117.3 (7)		
			
C3—N1—C2—C1	−0.36 (9)	N2—C1—C5—N3	−0.09 (9)
C5—C1—C2—N1	0.63 (9)	C2—C1—C5—C4	−0.38 (8)
N2—C1—C2—N1	−177.64 (5)	N2—C1—C5—C4	177.85 (5)
C2—N1—C3—C4	−0.16 (9)	C3—C4—C5—N3	177.88 (6)
N1—C3—C4—C5	0.40 (9)	C3—C4—C5—C1	−0.13 (9)
C2—C1—C5—N3	−178.32 (6)		

### Hydrogen-bond geometry (Å, °)

**Table:**

*D*—H···*A*	*D*—H	H···*A*	*D*···*A*	*D*—H···*A*
N1—H1N1···O2^i^	0.874 (13)	2.001 (13)	2.7750 (7)	147.0 (12)
N2—H1N2···O5	0.935 (14)	2.105 (15)	2.9070 (8)	143.1 (12)
N2—H1N2···O2^ii^	0.935 (14)	2.211 (14)	2.7767 (7)	118.1 (11)
N2—H2N2···O3^iii^	0.881 (14)	2.193 (14)	3.0006 (7)	152.3 (12)
N2—H2N2···O3^iv^	0.881 (14)	2.482 (14)	2.9231 (7)	111.6 (11)
N3—H1N3···O4	0.844 (16)	2.054 (16)	2.8653 (8)	161.0 (14)
N3—H2N3···O6^v^	0.827 (12)	2.130 (12)	2.9442 (7)	168.0 (12)
N2—H3N2···O5^iv^	0.871 (12)	1.963 (12)	2.8227 (8)	169.0 (12)
N2—H3N2···O6^iv^	0.871 (12)	2.494 (12)	3.1217 (7)	129.5 (10)
C2—H2···O3^iii^	0.910 (11)	2.439 (11)	3.0489 (8)	124.6 (9)
C2—H2···O1^vi^	0.910 (11)	2.552 (11)	3.1834 (8)	127.0 (9)
C2—H2···O3^iv^	0.910 (11)	2.570 (11)	3.1277 (8)	120.2 (9)
C3—H3···O6^i^	0.979 (12)	2.253 (12)	3.1170 (8)	146.6 (10)
C4—H4···O4^v^	0.926 (12)	2.559 (12)	3.4274 (8)	156.3 (10)

Symmetry codes: (i) *x*, *y*+1, *z*; (ii) *x*, −*y*+1/2, *z*−1/2; (iii) −*x*, *y*+1/2, −*z*+1/2; (iv) *x*, −*y*+1/2, *z*+1/2; (v) −*x*+1, *y*+1/2, −*z*+1/2; (vi) −*x*, −*y*+1, −*z*+1.
